# Prediction of daily childhood asthma exacerbation from ambient meteorological, environmental risk factors and respiratory viruses, Philadelphia, PA, 2011 to 2016

**DOI:** 10.1007/s11356-025-36089-w

**Published:** 2025-02-19

**Authors:** Wanyu Huang, Lucy F. Robinson, Amy H. Auchincloss, Leah H. Schinasi, Kari Moore, Steven Melly, Christopher B. Forrest, Chén C. Kenyon, Anneclaire J. De Roos

**Affiliations:** 1 Department of Epidemiology and Biostatistics, Dornsife School of Public Health, Drexel University, 3215 Market St, Philadelphia, PA 19104 USA; 2 Urban Health Collaborative, Dornsife School of Public Health, Drexel University, Philadelphia, PA USA; 3 Department of Environmental and Occupational Health, Dornsife School of Public Health, Drexel University, Philadelphia, PA USA; 4 The Applied Clinical Research Center, Children’s Hospital of Philadelphia, Philadelphia, PA USA; 5 PolicyLab, Children’s Hospital of Philadelphia, Philadelphia, PA USA

**Keywords:** Children, Asthma, Asthma exacerbation, Environmental risk factors, Aeroallergen season, ARIMA model, Prediction

## Abstract

Childhood asthma exacerbation has multiple risk factors that occur concurrently in the environment — including extreme meteorological conditions, air pollution, aeroallergens, and respiratory virus infections. Few studies have predicted asthma exacerbation based on multiple time-varying environmental risk factors, together. In this study, we constructed an autoregressive integrated moving average (ARIMA) model to predict “high-risk” days for childhood asthma exacerbation in Philadelphia, PA from 2011 to 2016, during the aeroallergen season of each year, using a total of 28,540 asthma exacerbation case events identified from electronic health record (EHR) data. We selected predictors from quantile weighted sum regression (gQWS), incorporating temporal lags and season-stratification (early- vs. late-season), which were entered subsequently into multivariable ARIMA models. We found that daily nitrogen dioxide (NO_2_), as well as monthly rhinovirus and respiratory syncytial virus (RSV) infection levels, were higher on the predicted “high-risk” days, as compared to days with lower childhood asthma exacerbation risk. The model performed better for late-season asthma exacerbation (July to October) than for early season (March to June). Future work and continued research is needed to facilitate local health guidelines pertaining to childhood asthma exacerbation.

**Supplementary Information:**

The online version contains supplementary material available at 10.1007/s11356-025-36089-w.

## Introduction

Childhood asthma exacerbation is associated with a large number of known environmental risk factors (Milligan et al. 2016) — including air pollutants (e.g., fine particulate matter, NO_2_), aeroallergens (i.e., pollens, molds) (De Roos et al. 2020; Fu and Tsai 2014), and meteorological factors (e.g., extreme temperature (Schinasi et al. 2022; Mireku et al. 2009; Stern et al. 2020) and heavy precipitation (Schinasi et al. 2020)). Respiratory virus infections, such as rhinovirus, are also known triggers of exacerbation (Zheng et al. 2018), for children with non-allergic asthma (Just et al. 2017). In urban settings, these risk factors can occur simultaneously; therefore, understanding risk conditions for asthma exacerbation on any particular day may require consideration of multiple allergic and non-allergic triggers, together. Developing risk profiles for childhood asthma exacerbation with prediction models is important, considering the co-existence of multiple exposures.

Studies to date have explored the effect of interactions between ambient pollutants (fine particulate matter (Cakmak et al. 2012; Guilbert et al. 2018) and nitrogen dioxide (NO_2_) (Chauhan et al. 2003)) and multiple types of aeroallergens (pollen from trees (Cakmak et al. 2012), grass (Guilbert et al. 2018; Murray et al. 2006), and weeds (Gleason et al. 2014)); and molds (Huang et al. 2023)) on asthma exacerbation, with some results supporting synergistic interactions for multiple risk factors. Further, results from a small number of studies have reported that environmental triggers of asthma may be stronger when children have respiratory virus infections; for example, associations between grass pollen and risk of asthma exacerbation were stronger among children with respiratory virus infections (Murray et al. 2006) (Schinasi et al. 2024). Despite evidence for potential interactions, the majority of epidemiologic studies to date have focused on non-interacting relationships of environmental exposures and viruses with childhood asthma exacerbation. A complicating issue in studying such interactions is that the effects of the triggers on asthma exacerbation are frequently lagged following exposure; therefore, interaction modeling requires consideration of appropriate exposure lags to best characterize the interactions. For example, epidemiologic studies have reported higher rates of asthma exacerbation during a time frame of up to 1-week following episodes of extreme temperature (Schinasi et al. 2022), air pollution (Orellano et al. 2017), and pollen (De Roos et al. 2020; Erbas et al. 2018). Correspondingly, when identifying risk factors for asthma exacerbation, it is important for related prediction strategies to consider exposure on relevant lag days. According to a work group report by the American Academy of Allergy, Asthma and Immunology (AAAAI) in 2019, foreseeable weather and climate changes can potentially increase the level of aeroallergens; and weather patterns also interact with the effects of air pollution — therefore affecting asthma health (Poole et al. 2019).

In order to further knowledge about high-risk environmental conditions for asthma exacerbation with consideration of potential interactions, and to potentially facilitate the decision-making process with regard to related health recommendations for local asthma management, we constructed autoregressive integrated moving average (ARIMA) models for daily rates of childhood asthma exacerbation based on multiple time-varying environmental predictors outdoor, in Philadelphia, PA. Our goal was to construct a model with outdoor risk factors (triggers) and respiratory viral infections comprised of multiple independent effects and interactions, that would be able to predict higher-than-normal rates of childhood asthma exacerbation (“high-risk” days), with a reasonable level of practicality and feasibility. Therefore, we included in the study a combination of selected predictors identified as strongly associated with asthma exacerbation in an ARIMA model. In identifying the “high-risk” days, we also considered potential interaction effects between predictors. We hypothesized that levels of environmental and viral risk factors would be higher, overall, on predicted “high-risk” days of childhood asthma exacerbation as compared to other days.

## Methods

### Study population and study period

The study population included children who were younger than 18 years old at the time of a patient visit within the Children’s Hospital of Philadelphia (CHOP) health system during the study period, including children with asthma who lived in Philadelphia, PA, from 2011 to 2016. Among these children, each asthma exacerbation “case” was identified as a clinical encounter in any of the CHOP clinical care settings (outpatient clinic, emergency department, and inpatient) that resulted in an asthma diagnosis (ICD-9: 493.00–493.99; ICD-10: J45) and a prescription for a systemic steroid for the same encounter. Specific encounters occurring within a single week (< 7 days) by the same child were excluded, by retaining the most “severe” case as inferred by the encounter setting (i.e., inpatient > ED > outpatient, as the preference). This study focused on asthma exacerbation prediction in Philadelphia, PA, during a defined period of the aeroallergen season (March 18 to October 30) in each year from 2011 through 2016, as these were the dates with aeroallergen measurement data available in each year. In the following, we refer to the aeroallergen seasons from 2011 through 2016 as the “study period” — with March 18 to June 30 as the “early season,” and July 1 to October 30 as the “late season.”

### Respiratory virus data

We used monthly counts of *respiratory virus infections* from clinical testing through CHOP’s emergency department (ED) and satellite sites to indicate infection rates in the population. The respiratory viruses of interest included rhinovirus, respiratory syncytial virus (RSV), influenza-A, influenza-B, coronavirus (non-COVID 19), parainfluenza viruses 1/2/3, and human metapneumovirus.

### Environmental data

Environmental predictors included *meteorological variables*, *air pollutants*, and *aeroallergens*.

We obtained *meteorological data* from the National Oceanic and Atmospheric Administration (NOAA), including daily average dry bulb temperature, relative humidity, wind speed, and precipitation. These data were obtained from 14 weather stations in total in the greater Philadelphia metropolitan area, including 5 stations within 20 km of Philadelphia, PA (NOAA 2017). For *air pollutants*, we used data from the EPA Air Quality System (AQS), including daily average PM_2.5_ (µg/m^3^), nitrogen dioxide (NO_2_) (ppb), and sulfur dioxide (SO_2_) (ppb), and daily maximum 8-h ozone (O_3_) (ppm). By 2015, there were 8 PM_2.5_, 4 ozone (O_3_) monitors, 4 NO_2_, and 2 SO_2_ monitors, respectively, as located in Philadelphia, PA, from where we retrieved outdoor air monitoring levels (USEPA 2017). Meteorological and air pollutant data were individually assigned to each child in the study population based on the monitor closest to their residential location at the time of the clinical encounter. For each child, daily measures of air pollutants were considered missing, given no valid measure from a monitor within 40 km of their residence on that day. Then, population-wide levels of meteorological and air pollutant predictors were summarized for each day of the study period by averaging individually assigned daily exposure levels for all children with asthma diagnoses (with- and without-exacerbations), which allowed us to estimate the daily air pollutant exposures as pertinent to our study population (i.e., both exacerbation and non-exacerbation asthma cases).

*Aeroallergen predictors* were represented by daily air concentrations, measured by one single regional National Allergy Bureau (NAB)-certified pollen and mold monitor (counter) (The Asthma Center, Philadelphia), located in downtown Philadelphia — with a median distance of 6.1 km, for all the asthma exacerbation cases ranged 0.2 to 40 km from the monitor. Aeroallergens were measured during the pollen season of each year, including tree pollen (total/oak/birch), grass pollen, weed pollen (total/ragweed), and molds. Levels of aeroallergens were measured on most weekdays; and accordingly, the days without original measuring levels (including measurement days considered extreme outliers) were imputed by linear interpolation. The sampling process for pollen grains/mold spores and a more detailed definition of extreme outliers were described in De Roos et al. (2020), one of our previous studies that considered the same study population in Philadelphia, PA (De Roos et al. 2020).

### Statistical analysis

In this study, we constructed ARIMA prediction models to predict daily asthma exacerbation rates. Daily rates were approximated by counts, assuming the pediatric population in Philadelphia remained relatively constant over the timeframe of the entire study period. Further, we identified environmental factors co-occurring on “high-risk” days for childhood asthma exacerbation. A priori, we defined “high-risk." days as days when daily childhood asthma exacerbation counts exceeded the 80th percentile over the entire study period, including the early- and late-seasons. Correspondingly, the rest of the days were defined as “lower-risk” days. We defined days from 2011 to 2015 as the training set, and year 2016 as the testing set.

### Univariate ARIMA model

Prediction models for daily childhood asthma exacerbation were constructed within the autoregressive integrated moving average (ARIMA) framework (Shekhar and Williams 2007), a common statistical framework for constructing prediction models with time series data, with the consideration of dependencies for observations (instead of taking each daily observation independently). We adopted a multi-stage approach for building the predictive model. To determine the dependency structure across days of daily asthma exacerbation, we first constructed a univariate ARIMA model (i.e., the base model) by decomposing the asthma exacerbation count time series into different components, including long-term trend, seasonal component, and a residual component unexplained by either long-term trend or seasonality. After determining the ARIMA parameters, viral and environmental variables were added to the model as predictors of the residual variation. For more details, see Supplementary Method Text for “ARIMA model – parameterization” section.

### Predictor selection for multivariable ARIMA model

As potential predictors, environmental data were linked to childhood asthma exacerbation events according to the following lags: no lag (same-day exposure, denoted “lag0”), and cumulative average levels lagged up to 5 days before the childhood asthma exacerbation event (lag01, lag02, lag03, lag04, lag05), as the majority of previous epidemiologic studies examined the effects of outdoor environmental exposures (air pollutants, aeroallergens) for up to a week preceding the asthma exacerbation events (Erbas et al. 2018; Orellano et al. 2017). We defined the “early season” as March 18 to June 30, and “late season” as July 1 to October 18 for aeroallergens (De Roos et al. 2020). And within both the “early” and “late” seasons, we applied generalized quantile weighted sum regression (gQWS) to select predictors to be entered into multivariable ARIMA models. gWQS is a generalized linear model framework in which an outcome of interest (e.g., asthma exacerbation) is regressed upon a set of predictors (e.g., environmental exposures) to determine the relative importance (i.e., weights) of each predictor, allowing the assessment of their relative contributions and their potential interactions (Carrico et al. 2015). These models can outperform individual regression and regularization methods (e.g., lasso, elastic nets), especially when correlations between the predictors are high (Carrico et al. 2015). For more detail regarding the application of gQWS framework in this study, see the Supplementary Methods Text.

### Multivariable ARIMA model

We used the selected predictors from gQWS models to fit ARIMA multivariable prediction models. In the multivariable models, we included day-of-week as indicator variables, in addition to the time and seasonal trends that were described by the univariate ARIMA model. More specifically, we fitted several models with different predictors: (1) Seasonal variables only (i.e., time trend, seasonal trend, day-of-week); (2) seasonal variables and virus infections; (3) seasonal variables, virus infections, and aeroallergens; (4) seasonal variables, virus infections, aeroallergens, meteorological and air pollutant variables.

### Assessing predictive performance

To assess the predictive performance of each model, we computed the root mean squared error (RMSE) for prediction of asthma rates from the training (2011 to 2015) and testing (2016) sets. In addition, we calculated the percentage of correctly predicted “high-risk” days (# correctly predicted/ # true “high-risk” days × 100%), as well as sensitivity and specificity of this classification, in both the testing and training sets. “High-risk” days had predicted counts of childhood asthma exacerbations above 80% over the study period. The remaining days were taken as “lower-risk” days.

We then used data from the entire study period (2011 to 2016 — i.e., “training” and “testing” sets) and re-fit the ARIMA models that best described “high-risk” days during the early/late season and used the smallest number of predictors. Additionally, we summarized the distribution of corresponding environmental and virus predictors on “high-risk” days compared to lower-risk days.

The statistical analysis was conducted using R (version 4.1.2), with ARIMA models constructed using the “forecast” package, and generalized weighted quantile sum (qWQS) regression for selection of predictor variables using the “gWQS” package.

## Results

### Univariable time series and base model

In the decomposed univariable time series for childhood asthma exacerbation counts, we observed a recurring seasonal pattern with peaks in the spring and winter seasons and lower daily counts in summer (Supplementary Fig. 1).

The median of asthma exacerbation counts was 19/day (interquartile range: 13/day to 27/day) within the study population (Table 1, shown for aeroallergen sampling dates March 18 to October 30, during 2011 to 2016). Percentile distributions for all predictors — including daily levels of meteorological factors, air pollutants, and aeroallergens, as well as counts of monthly virus infections are also shown in Table 1. Levels of PM_2.5_ had a median of 8.7 µg/m^3^ (IQR: 6.6 – 11.7 µg/m^3^), and levels of NO_2_ had a median of 23.9 ppb (IQR: 18.3 – 30.5 ppb). Among all types of pollen, tree pollen levels exhibited the largest interquartile range (0 – 141.1 grains/m^3^) with a median of 4.2 grains/m^3^, compared to pollens from grass and weeds (total weed and ragweed). The median level of molds was 3397.0 spores/m^3^ (IQR: 1895.1 – 4416.2 spores/m^3^). Among respiratory viruses, monthly detected rhinovirus infections exhibited the highest median (120/month) and the largest interquartile range (88/month – 169/month), followed by respiratory syncytial virus (RSV), influenza-A and B viruses.

**Table 1:** Percentile distributions of daily childhood asthma exacerbation counts and environmental/viral predictor levels during the pollen season (March 18 to October 30), in Philadelphia, PA, 2011 to 2016

Percentiles	p5	p25	p50	p75	p90	p95	p99
Exacerbation case	3	13	19	27	35	40	77
Temperature (°C)	7.1	15.1	20.9	24.8	27.4	28.5	30.4
Relative humidity	40.6	54.8	63.7	73.1	81.3	85.4	90.0
Precipitation (inch)	0.0	0.0	0.0	0.1	0.4	0.8	2.1
Wind (mph)	3.7	5.5	7.1	9.1	11.4	12.9	15.9
PM_2.5_ (µg/m^3^)	4.3	6.6	8.7	11.7	15.6	17.5	24.4
Ozone (O_3_) (ppm)	0.020	0.032	0.040	0.049	0.059	0.064	0.076
Nitrogen dioxide (NO_2_) (ppb)	11.8	18.3	23.9	30.5	37.4	41.8	53.2
Sulfur dioxide (SO_2_) (ppb)	0.7	1.5	2.3	3.5	5.1	6.3	8.3
Tree pollen, total (grain/m^3^)	0.0	0.0	4.2	141.1	477.9	775.7	1436.6
Grass pollen (grain/m^3^)	0.0	0.0	4.1	8.5	23.2	33.8	52.7
Weed pollen, total (grain/m^3^)	0.0	4.2	10.3	20.5	40.2	60.6	106.1
Ragweed pollen (grain/m^3^)	0.0	0.0	0.0	4.2	20.5	33.1	60.9
Molds (spore/m^3^)	723.9	1895.1	3397.0	4416.2	5517.5	6119.2	8757.2
Rhinovirus	62.0	88.0	120.0	169.0	227.0	241.0	256.0
Respiratory syncytial virus	2.0	4.0	8.0	15.0	38.0	57.0	142.2
Influenza-A (IFV-A)	0.0	0.0	0.0	3.0	24.0	36.0	128.0
Influenza-B (IFV-B)	0.0	0.0	1.0	6.0	27.0	44.0	92.1

We selected ARIMA (0,2,2) as the base model (for both early- and late-season), as it had the smallest testing root mean squared error among all models with the highest percentage of successfully predicted high-risk days.

### Predictors selected for ARIMA models from gQWS

**Fig. 1:**
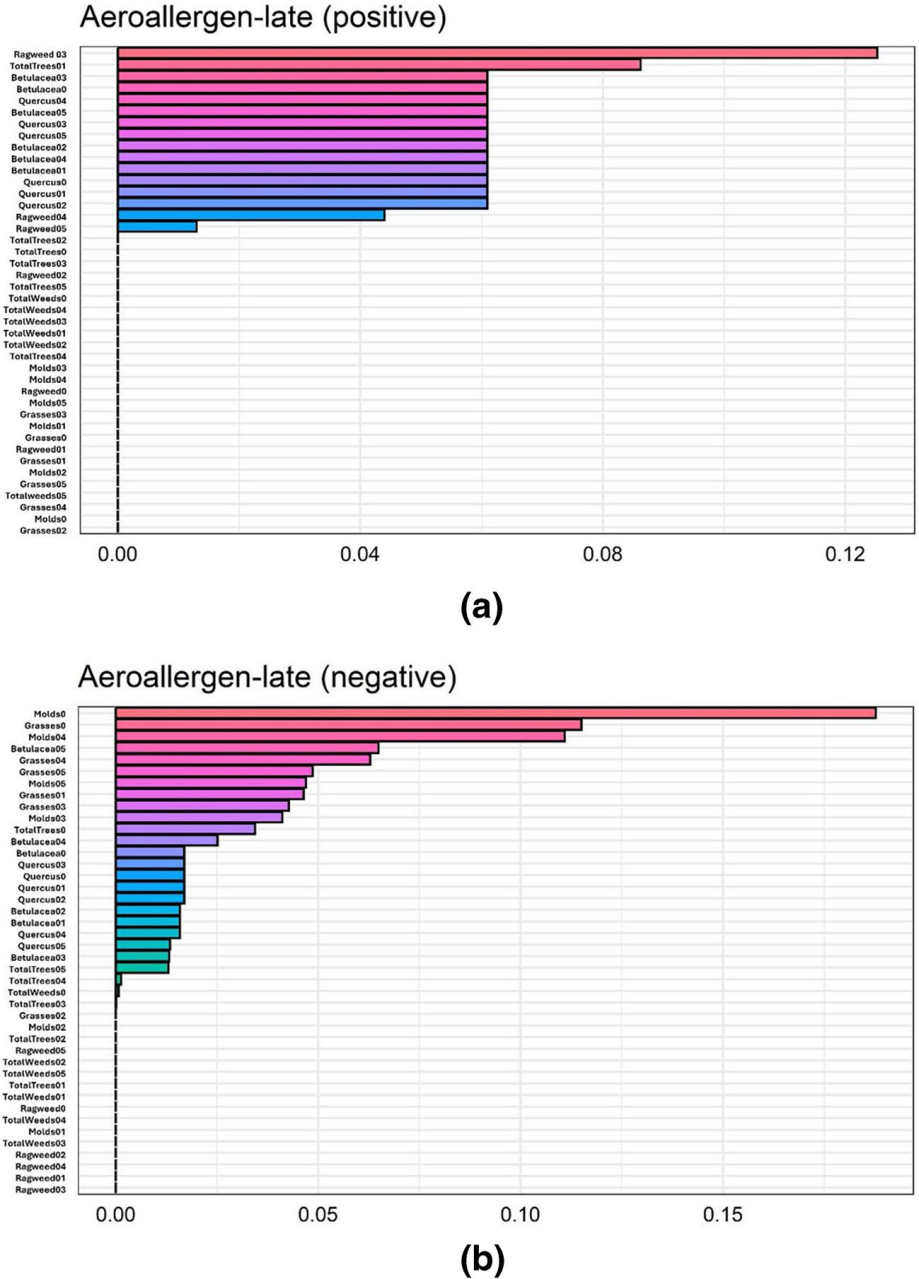
**a** Relative importance of predictors of late-pollen season prediction model, as indicated by weights estimated from the generalized quantile weighted sum (gQWS) model, among predictor variables of aeroallergen predictors (overall positive relationship with daily childhood asthma exacerbation counts)**. b** Relative importance of predictors within the late pollen season, as indicated by weights estimated from the generalized quantile weighted sum (gQWS) model, among predictor variables of aeroallergen predictors (overall negative relationship with daily childhood asthma exacerbation counts)

**Fig. 2:**
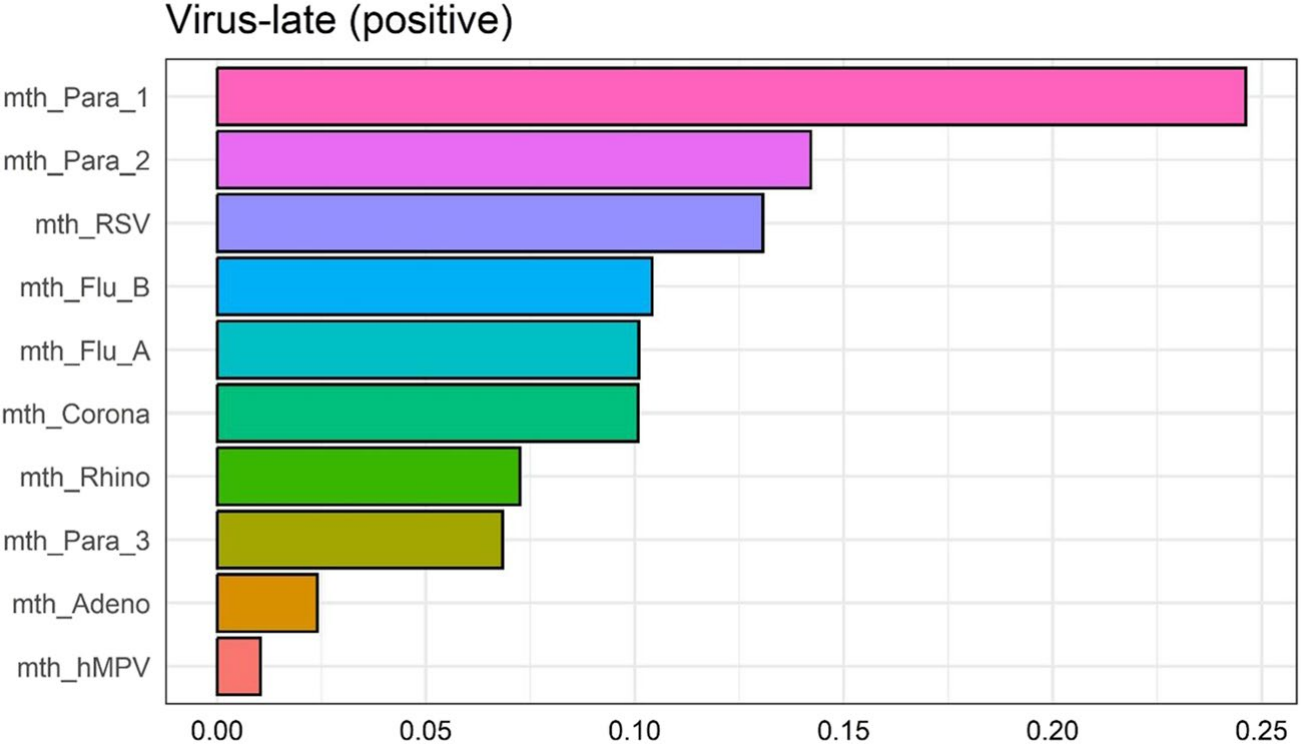
Relative importance of predictors of late-season prediction models, as indicated by weights estimated from the generalized quantile weighted sum (gQWS) model, among predictor variables of viral infection counts factors (overall positive relationship with daily childhood asthma exacerbation counts)

Predictors to be entered into multivariable ARIMA models for the early- (Supplementary Table 4) and late-season (Table 2) were selected from the gQWS models, based on the relative importance (weights) of each predictor. The relative importance of the predictors was shown in Figs. 1 and 2 and Supplementary Figs. 2/3, including those in each group of environmental risk factors (i.e., respiratory virus infections, meteorological factors, air pollutants, aeroallergens). Below, we refer to any predictor within a gQWS model fitted with the assumption of an overall positive relationship (at a specific cumulative lag) as a “positive predictor;” we refer to any predictor fit with the assumption of an overall negative relationship as a “negative predictor” (see Supplementary material for more details).

**Table 2:** Selected predictors (meteorological factors, air pollutants, aeroallergens, detected respiratory virus infections), determined based on 80% of cumulative weight from the gWQS regression, within the late pollen season (July 1 to Oct 30) from each study year (2011 to 2016)

Predictor type	Predictor (lag)	lag0	lag01	lag02	lag03	lag04	lag05
Meteorological^a^	Temperature	X(-)				X(-)	X(-)
	Relative humidity						
	Wind					X(-)	
	Precipitation						X(-)
Air pollutants^a^	PM_2.5_				X(-)		X(-)
	O_3_		X(-)			X(-)	X(-)
	NO_2_						
	SO_2_						
Aeroallergens^a^	Tree pollen, total	X(-)	X( +)		X( +)		
	Oak pollen	X( +)	X( +)		X( +)	X( +)	X( +)
	Birch pollen	X( +)	X( +)	X( +)		X( ±)	X( ±)
	Grass pollen	X(-)	X(-)		X(-)	X(-)	X(-)
	Weed pollen, total						
	Ragweed pollen				X( +)		
	Molds	X(-)			X(-)	X(-)	X(-)
Respiratory virus^b^	CORONA	X( +)	N/A	N/A	N/A	N/A	N/A
	RHINO	X( +)	N/A	N/A	N/A	N/A	N/A
	PARA1	X( +)	N/A	N/A	N/A	N/A	N/A
	PARA2	X( +)	N/A	N/A	N/A	N/A	N/A
	PARA3		N/A	N/A	N/A	N/A	N/A
	ADENO		N/A	N/A	N/A	N/A	N/A
	RSV	X( +)	N/A	N/A	N/A	N/A	N/A
	IFV-A	X( +)	N/A	N/A	N/A	N/A	N/A
	IFV-B	X( +)	N/A	N/A	N/A	N/A	N/A
	hMPV						

X: selected predictor; XX: forced-in selected predictor

Within parentheses following each selected predictor, “ + ”/ “-” indicates that the predictor was within a “mixture” with overall positive/negative relationship to childhood asthma exacerbation

^a^Meteorological, air pollutant, and aeroallergen predictors were all daily-level variables (with daily-level cumulative lags)

^b^Only same-month respiratory virus predictors were considered (with rest of the cells indicated as N/A's within the table)

During the early aeroallergen season (March 18 to June 30) (Supplementary Table 4), respiratory virus infections were positive predictors, and air pollutants/aeroallergens were either positive or negative predictors. More specifically, among the respiratory virus infections, monthly parainfluenza 3 counts had the highest predictor weight, followed by monthly levels of influenza virus-A and influenza virus-B. Among all potential air pollutants, NO_2_ (lag0, lag05) captured most of the cumulative weight as a positive predictor, followed by SO_2_ (lag05, lag01), whereas PM_2.5_ (lag03, lag04, lag05) and O_3_ (lag0, lag01, lag05) were both shown as negative predictors. Among the aeroallergens, same-day tree pollen captured more than 40% of the cumulative weight as a positive predictor, followed by same-day and lag02 birch tree pollen, whereas molds (lag0, lag01, lag02, lag05) and ragweed (lag0 to lag05) were shown as negative predictors.

During the late season (July 1 to October 18), respiratory virus infections were again shown as positive predictors, with the highest weights observed for monthly infection counts of parainfluenza 1/2 and respiratory syncytial virus (RSV) (Fig. 2). For aeroallergens, ragweed pollen (lag03) exhibited the highest weight as a positive predictor, followed by total tree pollen (lag01) and birch pollen predictors (lag0 and lag05), whereas same-day molds and grasses were both negative predictors (Fig. 1a, b). Air pollutants were only able to be modeled as negative predictors, among which PM_2.5_ (lag03, lag05) and O_3_ (lag02, lag05) captured most of the weights.

Last, during both the early and late season, gQWS models were fit under the assumption that meteorological factors, as an entire predictor group, exhibited an overall negative association with daily asthma exacerbation counts. Daily average temperature (lag04, lag05) accounted for most of the cumulative weights among all meteorological predictors — especially during the late aeroallergen season.

### ARIMA prediction model

Overall, in the early season, prediction models had 100 percent specificity but low sensitivities (≤ 10%) (Supplementary Table 1a). For this reason, we only present late-season models in detail (Table 3). During the late aeroallergen season, the best model had 4 virus predictors (respiratory syncytial virus [RSV], human metapneumovirus [hMPV], parainfluenza 2, and parainfluenza 3), as well as multiple aeroallergen predictors (ragweed pollen, total tree pollen, oak tree pollen, birch pollen at different lags). The model had a test root mean squared error (RMSE) of 10.80, as well as a sensitivity of 0.96 in capturing the high-risk asthma exacerbation days. In comparison, the base model (model considering time trends, seasonal trends, with the adjustment of day-of-week), had a testing RMSE of 11.09 and a sensitivity of 0.89. Sensitivities and specificities were calculated based on an 80% cutoff of predicted asthma exacerbation daily counts.

**Table 3:** A series of ARIMA (0,2,2) models constructed within the late pollen season (July 31 to October 30), with different sets of predictors. Root mean squared errors (RMSE) were reported from both training (2011 to 2015) and testing (2016) datasets. Percentages of correctly predicted high-risk days, as well as sensitivity (SN) and specificity (SP) were reported

Predictors	Training RMSE	Testing RMSE	Percent high-risk	SN	SP
Viruses^a^	5.90	10.98	96.0	0.96	0.57
Viruses^a^ + aeroallergens^b^	5.85	10.80	96.0	0.96	0.57
Viruses^a^ + aeroallergens^b^ + temperature + O3	5.65	8.44	32.0	0.32	0.95
Viruses^a^ + aeroallergens^b^ + viruses × ragweed pollen^c^	5.79	12.09	64.0	0.64	0.536
Viruses^a^ + aeroallergens^b^ + viruses × tree pollen^d^	5.79	11.60	96.0	0.96	0.588

^a^Viruses: RSV (respiratory syncytial virus) + hMPV (human metapneumovirus) + para2 (parainfluenza virus 2) + para3 (parainfluenza virus 3)

^b^Aeroallergens: a subset of aeroallergen predictors positively associated with childhood asthma exacerbation, leading to model convergence with virus predictors, including: *ragweed pollen (lag 03), tree pollen (total) (lag 01), birch pollen (lag 03), birch pollen (lag 0), oak tree pollen (lag 04), birch pollen (lag05), oak tree pollen (lag 05), birch pollen (lag 02), birch pollen (lag 04), birch pollen (lag 01)*

^c^Ragweed pollen: lag03

^d^Tree pollen: lag01

#### Single predictor models

To construct the prediction models, we started with a subset of virus and aeroallergen predictors — as there were a low number of days with co-presence of high-viral infection and high-aeroallergen days. Among models with a single virus predictor, four models exhibited a sensitivity of > 80% in predicting “high-risk” days: respiratory syncytial virus (RSV), human metapneumovirus (hMPV), parainfluenza 2, and parainfluenza 3. The single predictor model using parainfluenza 3 virus exhibited the lowest testing root mean squared error (RMSE) (10.00), as well as the highest sensitivity (0.96) and specificity (0.71) among the four single-predictor models (Supplementary Table 1a/b).

#### Main effects models

We included four virus predictors (human metapneumovirus [hMPV], parainfluenza 2, and parainfluenza 3) into the main effect model. In addition, a subset of aeroallergen predictors was added into the model, including total tree pollen, ragweed pollen, oak pollen, and birch pollen variables at different lags (Table 2, Fig. 1a). After adding aeroallergen predictors, the model with virus and aeroallergen predictors had a testing RMSE of 10.80 — lower than the RMSE of most models with the individual virus predictors except for the model with parainfluenza 3 as the predictor. Further, to the model described above, we selected and added temperature (lag05) and ozone (lag05) to the model described above — despite both meteorological and air pollutant predictors exhibiting overall negative relationships with childhood asthma exacerbation during the late aeroallergen season (Supplementary Figs. 2, 3). After the addition of the meteorological and air pollutant predictors, the testing RMSE was further lowered, and the percentage of predicted “high-risk” days (sensitivity) dropped considerably, to 30%, compared to when temperature and air pollutant predictors were not added to the model (96%).

#### Interaction models

Next, we entered interaction terms into the main effect model with aeroallergen and virus predictors and identified which predicted values from the test set were able to best capture the “high-risk” days (based on sensitivity and root mean squared error). When interaction terms were included for virus and total tree pollen predictors, percentage predicted “high-risk” days were same to reduced models with only tree pollen and viruses as the single predictor model independent predictors. When interaction terms were included for viral and ragweed predictors, the percentage predicted “high-risk” days decreased to 64% — lower than when modeling the ragweed pollen and the viruses as independent predictors.

### Distribution of predictors: high-risk vs. lower-risk days

In the final stage of this work, we refitted the model with aeroallergen and virus predictors, *without* interaction terms. This was the most parsimonious model that identified the highest percentage of “high risk” days. The model was fitted within the late aeroallergen seasons of the study period, during the years from 2011 to 2016. From that model, we obtained the distributions of all same-day viral and environmental factors, on the predicted “high-risk” days (> 80th percentile childhood asthma exacerbation counts) (Supplementary Table 2) — in comparison with the remaining “lower-risk” days (≤ 80th percentile) (Supplementary Table 3). For respiratory viruses, the predicted “high-risk” scenarios captured the days with the higher monthly detected rhinovirus (median: 151/month) and respiratory syncytial virus (median: 8/month) infection counts. Daily temperature was lower on “high-risk” days (median: 19.3 °C, interquartile range [IQR]: 14.9 °C – 24.1 °C), compared to “lower-risk” days (median: 23.9 °C, interquartile range [IQR]: 20.3 °C –26.5 °C). Distributions of the other meteorological factors were similar between predicted “high-risk” days and the remaining days. For air pollutants, NO_2_ levels were higher on “high-risk” days (median: 24.4 ppb; interquartile range [IQR]: 20.7 – 31.4 ppb) than on “lower-risk” days (median: 21.3 ppb; interquartile range [IQR]: 16.8 – 27.7 ppb). Levels of most aeroallergens did not exhibit substantial differences between “high-risk” and “lower-risk” days. For example, mold levels were similar on high-risk days (median: 3787.8 spores/m^3^; IQR: [2886.5 – 4687.8 spores/m^3^]) and lower-risk days (median: 3959.3 spores/m^3^; IQR: [3305.1 – 4764.5 spores/m^3^]). Yet, aeroallergens including grass and weed pollen (including ragweed, and total) had somewhat lower median levels on predicted “high-risk” days — for example, ragweed pollen (high-risk day IQR: 0 – 10.5 grain/m^3^; low-risk day IQR: 0 – 15.7 grain/m^3^).

## Discussion

Under the multivariable ARIMA framework, we incorporated multiple environmental predictors of daily childhood asthma exacerbation, along with potential interactions between different predictor types. Therefore, the framework was able to better represent “real-world” exposure scenarios as related to risk of asthma exacerbation (Milligan et al. 2016), as compared to most of the past studies that addressed the independent effect of each individual risk factor. With the consideration of multiple environmental risk factors and their potential interactions, we found that “high-risk” asthma exacerbation days were the days when both aeroallergens and rhinovirus/respiratory virus infection counts were high, during the late aeroallergen season.

NO_2_ and virus infection variables were good predictors of high-risk exacerbation days under the ARIMA framework, during the late season. Although some aeroallergens predictors (e.g., ragweed, tree pollen) were identified as more important predictors in the model, NO_2_ levels exhibited greater differences between the predicted “high-risk” and “lower-risk” days. In particular, concentrations of NO_2_ and rhinovirus/respiratory syncytial virus infection were substantially higher on the predicted “high-risk” asthma exacerbation days during the late season (Lucas and Platts-Mills 2005; Samoli et al. 2011).

Among all the air pollutant predictors we evaluated, NO_2_ had higher levels on predicted “high-risk” days. This was consistent with findings from a recent systematic review which suggested higher risk of childhood asthma exacerbation upon short-term NO_2_ exposure (Zheng et al. 2021). In addition, past experimental evidence supported increased inflammatory response in pulmonary tissues and reduced immunity against respiratory viruses with NO_2_ exposure (Cisneros et al. 2021). Ragweed pollen exhibited lower levels on predicted “high-risk” days. This result was consistent with our previous study, conducted within the same study population as the current study, where an inverse association was observed for ragweed pollen in relation to odds of childhood asthma exacerbation (De Roos et al. 2020). Our results are also consistent with other epidemiologic studies of associations between ragweed pollen exposure and severe asthma exacerbation (Annesi-Maesano et al. 2023) — despite mechanistic studies suggesting that ragweed pollen can trigger allergic inflammatory response through multiple signaling pathways (Deng et al. 2019; Li et al. 2011). Last, temperatures were lower on predicted “high-risk” days. Epidemiologic evidence has suggested that cold temperature (Xu et al. 2018) and sudden drop of temperature (Zhu et al. 2022) are risk factors for asthma exacerbation, even in areas with subtropical climate. Possible mechanisms include that low temperature can potentially decrease lung function, suppress the immune system, and increase the survival of different respiratory viruses (Xu et al. 2018).

In this study, incorporating multiple independent risk factors (i.e., viruses, aeroallergens) improved the predictions of “high-risk” days, although the addition of interaction terms did not. A recent study also found that temporally varying environmental data improved prediction of childhood asthma exacerbations, together with individual patients’ clinical characteristics as obtained from electronic health records (Hurst et al. 2022). Despite suggestions from prior mechanistically focused research that interactions between aeroallergens and air pollutants could be important in predicting exacerbations (Knox et al. 1997; D’Amato et al. 2001), little epidemiologic evidence has been found for such interactions (Lam et al. 2021). In addition, we found little evidence for modification by respiratory virus infections on the effects of air pollutants and aeroallergens on childhood asthma exacerbation, in the same study population (Schinasi et al. 2024). Our prediction model exhibited good performance for predicting “high-risk” asthma exacerbation during the late season. During the “September epidemic” after children return to school, Johnston et al. (2005) observed that the percentage of children with respiratory virus detection, as a well-known important contributor to asthma exacerbation in the fall, was significantly higher for those with asthma exacerbation compared to non-exacerbation cases (Johnston et al. 2005). In addition, it has been observed that for inner-city children, sensitization to aeroallergens was important in predicting risk of asthma exacerbation during the fall season (Teach et al. 2015). Yet during the early season, our study showed low sensitivity in predicting high-risk asthma exacerbation days. According to Teach et al. (2015), aeroallergens were suggested to be more predictive for childhood asthma exacerbation, within the city during the fall season (compared to other seasons — i.e., spring, summer, winter) — as indicated by a higher portion of variance from the models explained by serum IgE levels (Teach et al. 2015). In addition, people are more likely to spend time exercising outdoors starting from the spring season, which could, by itself, be a trigger for asthma exacerbation (PennMedicine 2017). Yet, our model was not able to take account related lifestyle factors into consideration, which can be another possible explanation for the disparities of model performance between the early and the late season.

Our study is one of the first that incorporated multiple time-varying risk factors in the prediction of childhood asthma exacerbation risk — with potential consideration of exposure latencies and interactions between different types of environmental risk factors. By contrast, thus far, most of the prediction models for asthma exacerbation/asthma severity have focused on “personalized predictions” involving individual-level clinical factors (e.g., symptom, medication, genetic factors), but not environmental factors (Finkelstein and Jeong 2017; Xu et al. 2011). Our study also had the following limitations. First, the respiratory virus data were only obtained from clinical testing (not routine surveillance monitoring) modeled in counts at the monthly level, within the Philadelphia metropolitan area to approximate community-level infections; therefore, they were subject to measurement errors. Second, aeroallergen data were obtained from a single monitor at the Center City, and air pollutant estimates were obtained from the nearest monitors for children over the entire study period; therefore, spatial resolution could be improved in future studies. Of note, our study will mainly provide health implications for childhood asthma during the late season, due to the low sensitivity of model predictions during the early (spring) season. Besides, as our study population was identified from the City of Philadelphia, and the exposure data for each child over the study period were obtained from the city, this could potentially limit the generalizability of our results to other areas.

Nevertheless, we believe that our findings are able to provide important evidence, furthering the knowledge for health care professionals (including school nurses) in identifying and responding to “high-risk” days locally, in Philadelphia, PA, on which childhood asthma exacerbations (especially severe cases) are more likely to be induced (Hillemeier et al. 2006; Hanley Nadeau and Toronto 2016). So far, with additional evidence provided by our study that concerns the joint effects of multiple environmental risk factors, we aim to facilitate more comprehensive asthma guidelines. In addition, knowledge provided by our study could also facilitate decision-making processes, regarding the issuance of health advisories pertinent to a vulnerable population (i.e., asthmatic children residing within the city) — including recommendations for staying indoor, noticeably on days with both high respiratory viral infection rates and aeroallergen levels in the fall.

Together with previous works, our study suggests that continued efforts are still needed in improving outdoor air quality (Huang et al. 2021); as well as strengthening related local aeroallergen (e.g., pollen) monitoring networks (De Roos et al. 2020) for better asthma management in the city. Of note, children spend majority of their time at home, where multiple household-level indoor triggers of asthma exacerbations also co-exist — such as dust mites (Gautier and Charpin 2017) and indoor microbiomes (Fu et al. 2021; Fu et al. 2022). Hence, the implementation of health guidelines and recommendation of stay-at-home, can potentially be more effective when related indoor risk triggers are properly managed (Gautier and Charpin 2017). Last, future research could be conducted within limited time periods to better inform the public regarding season-specific risks and provide predictions.

## Supplementary Information

Below is the link to the electronic supplementary material.

Supplementary file1 (DOCX 968 KB)

## Data Availability

Data cannot be made available due to their containing information that could compromise the privacy of research participants.
