# Supplementary material for: Prediction of daily childhood asthma exacerbation from ambient meteorological, environmental risk factors and respiratory viruses, Philadelphia, PA, 2011 to 2016
Source: Environ Sci Pollut Res Int. 2025 Feb 19;32(10):6041–52. doi: 10.1007/s11356-025-36089-w (PMC11913910; doi:10.1007/s11356-025-36089-w)
Supplement: Supplementary file 1 — Supplementary file1 (DOCX 968 KB) [file 11356_2025_36089_MOESM1_ESM.docx]

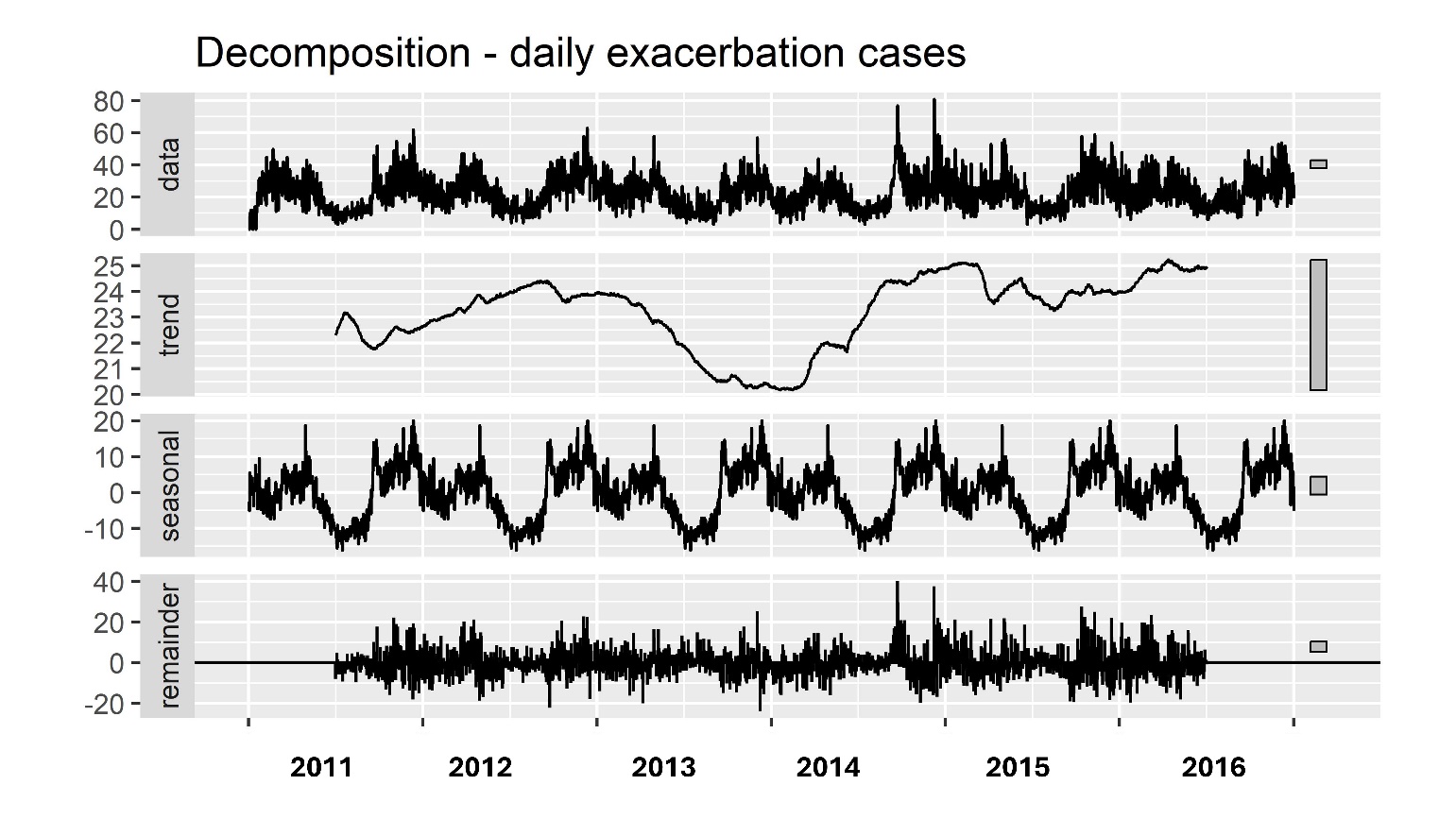


Data: the time series data for childhood asthma exacerbation; trend: long-term trend, seasonal: seasonal trend; remainder: a remaining part unexplained by time trend – including the variability by different environmental and viral predictor, as well as random errors. Note that scale for each section of the figure (data/trend/seasonal/reminder) was different.

Supplementary Fig.1. Decomposition of the time series for childhood asthma exacerbation, in Philadelphia, PA, from 2011 to 2016. Data were decomposed into long-term trend, seasonal trend, and a remaining part (unexplained by time trend).

Supplementary Table 1a. ARIMA (0,2,2) models in prediction of daily childhood asthma exacerbation counts, during the early pollen season (March 18 to June 30), with 2011 to 2015 as the training set, and 2016 as the testing set. Predictors included individual monthly respiratory counts variables. Root mean squared errors (RMSE), as well as percent ‘high-risk’ days predictors, and sensitivity (SN) and specificity (SP), were reported.

| **Predictors** | **Training RMSE** | **Testing RMSE** | **Percent high-risk** | **SN** | **SP** |
| --- | --- | --- | --- | --- | --- |
| Rhinovirus | 6.01 | 12.47 | 0.0 | 0.00 | 1.00 |
| Respiratory syncytial virus | 6.03 | 12.17 | 0.0 | 0.00 | 1.00 |
| Influenza virus A | 6.07 | 17.66 | 9.5 | 0.10 | 0.86 |
| Influenza virus B | 6.04 | 11.18 | 0.0 | 0.00 | 1.00 |
| Adenovirus | 6.07 | 12.28 | 0.0 | 0.00 | 1.00 |
| Human metapneumovirus | 5.99 | 13.03 | 0.0 | 0.00 | 1.00 |
| Parainfluenza virus 1 | 6.10 | 11.28 | 0.0 | 0.00 | 1.00 |
| Parainfluenza virus 2 | 6.13 | 14.43 | 0.0 | 0.00 | 1.00 |
| Parainfluenza virus 3 | 6.10 | 12.87 | 0.0 | 0.00 | 1.00 |
| All viruses^a^ | 5.97 | 11.64 | 0.0 | 0.00 | 1.00 |

Supplementary Table 1b. ARIMA (0,2,2) models in prediction of daily childhood asthma exacerbation counts, during the late pollen season (July 1 to October 30), with 2011 to 2015 as the training set, and 2016 as the testing set. Predictors included individual monthly respiratory counts variables. Root mean squared errors (RMSE), as well as percent ‘high-risk’ days predictors, and sensitivity (SN) and specificity (SP), were reported.

| **Predictors** | **Training RMSE** | **Testing RMSE** | **Percent high-risk** | **SN** | **SP** |
| --- | --- | --- | --- | --- | --- |
| Rhinovirus | 5.87 | 8.58 | 32.0 | 0.32 | 1.00 |
| RSV | 5.95 | 12.18 | 96.0 | 0.96 | 0.40 |
| Influenza virus A | 5.95 | 9.41 | 52.0 | 0.52 | 0.78 |
| Influenza virus B | 5.97 | 8.92 | 32.0 | 0.32 | 1.00 |
| Adenovirus | 5.89 | 10.46 | 40.0 | 0.40 | 0.70 |
| Human metapneumovirus | 5.97 | 12.04 | 84.0 | 0.84 | 0.46 |
| Parainfluenza virus 1 | 5.95 | 8.87 | 0.0 | 0.00 | 1.00 |
| Parainfluenza virus 2 | 5.97 | 12.25 | 96.0 | 0.96 | 0.41 |
| Parainfluenza virus 3 | 5.92 | 10.00 | 96.0 | 0.96 | 0.71 |
| All viruses^a^ | 5.87 | 8.74 | 44.0 | 0.44 | 0.95 |

^a^All viruses: rhinovirus + respiratory syncytial virus + influenza virus A + influenza virus B + adenovirus + human metapneumovirus + parainfluenza virus 1 + parainfluenza virus 2 + parainfluenza virus 3.

Supplementary Table 2. distribution of environmental/viral factors on predicted ‘high-risk’ days (asthma exacerbation counts > the 80th percentile), during the late pollen season (July 1 to October 30), within each year from 2011 to 2016, in Philadelphia, PA.

| **Predictor** | **p5** | **p25** | **p50** | **p75** | **p90** | **p95** | **p99** |
| --- | --- | --- | --- | --- | --- | --- | --- |
| Temperature (°C) | 10.3 | 14.9 | 19.3 | 24.0 | 27.1 | 28.2 | 29.5 |
| Relative humidity | 52.1 | 61.2 | 69.0 | 75.3 | 83.3 | 85.7 | 91.2 |
| Precipitation (inch) | 0.0 | 0.0 | 0.0 | 0.1 | 0.4 | 0.8 | 1.8 |
| Wind (miles/h) | 3.1 | 4.7 | 6.4 | 8.3 | 9.9 | 11.2 | 14.9 |
|  |  |  |  |  |  |  |  |
| PM2.5 (μg/m^3^) | 4.8 | 6.8 | 8.8 | 11.8 | 15.8 | 17.2 | 20.7 |
| Ozone (O_3_) (ppm) | 0.0 | 0.0 | 0.0 | 0.0 | 0.1 | 0.1 | 0.1 |
| Nitrogen dioxide (NO_2_) (ppb) | 14.7 | 20.7 | 24.4 | 31.4 | 37.1 | 40.5 | 54.1 |
| Sulfur dioxide (SO_2_) (ppb) | 0.7 | 1.4 | 2.2 | 3.1 | 4.8 | 6.2 | 7.5 |
|  |  |  |  |  |  |  |  |
| Tree pollen, total (grain/m^3^) | 0.0 | 0.0 | 0.0 | 2.1 | 4.2 | 6.3 | 11.1 |
| Oak tree pollen (grain/m^3^) | 0.0 | 0.0 | 0.0 | 0.0 | 0.0 | 0.0 | 0.0 |
| Grass pollen (grain/m^3^) | 0.0 | 0.0 | 2.1 | 4.1 | 4.4 | 6.4 | 19.0 |
| Ragweed pollen | 0.0 | 0.0 | 3.2 | 10.5 | 23.3 | 30.4 | 45.7 |
| Total weed pollen (grain/m^3^) | 2.2 | 6.5 | 14.5 | 26.1 | 40.5 | 70.8 | 111.8 |
| Birch pollen (grain/m^3^) | 0.0 | 0.0 | 0.0 | 0.0 | 0.0 | 0.0 | 0.0 |
| Molds (spore/m^3^) | 1810.3 | 2886.5 | 3787.8 | 4687.8 | 5805.3 | 6705.0 | 9406.3 |
|  |  |  |  |  |  |  |  |
| Rhinovirus | 58.0 | 88.0 | 151.0 | 164.0 | 227.0 | 256.0 | 256.0 |
| Respiratory syncytial virus | 3.0 | 4.0 | 8.0 | 15.0 | 16.0 | 19.0 | 19.0 |
| Influenza-A | 0.0 | 0.0 | 0.0 | 1.0 | 1.0 | 1.0 | 1.0 |
| Influenza-B | 0.0 | 0.0 | 0.0 | 1.0 | 1.0 | 1.0 | 3.0 |

Supplementary Table 3. distribution of environmental/viral factors on predicted ‘lower-risk’ days (asthma exacerbation counts <= the 80th percentile), during the late pollen season (July 1 to October 30), within each year from 2011 to 2016, in Philadelphia, PA.

| **Predictor** | **p5** | **p25** | **p50** | **p75** | **p90** | **p95** | **p99** |
| --- | --- | --- | --- | --- | --- | --- | --- |
| Temperature (°C) | 12.6 | 20.3 | 23.9 | 26.4 | 28.6 | 29.4 | 31.4 |
| Relative humidity | 49.2 | 58.2 | 65.4 | 72.9 | 79.9 | 85.5 | 91.0 |
| Precipitation (inch) | 0.0 | 0.0 | 0.0 | 0.1 | 0.5 | 0.9 | 2.7 |
| Wind (miles/h) | 3.2 | 5.1 | 6.6 | 8.6 | 10.3 | 11.8 | 15.2 |
|  |  |  |  |  |  |  |  |
| PM2.5 (μg/m^3^) | 4.3 | 7.0 | 9.3 | 12.2 | 16.3 | 18.7 | 25.9 |
| Ozone (O_3_) (ppm) | 0.0 | 0.0 | 0.0 | 0.1 | 0.1 | 0.1 | 0.1 |
| Nitrogen dioxide (NO_2_) (ppb) | 11.4 | 16.8 | 21.3 | 27.7 | 34.2 | 37.7 | 45.7 |
| Sulfur dioxide (SO_2_) (ppb) | 0.6 | 1.4 | 2.1 | 3.3 | 4.8 | 6.1 | 9.2 |
|  |  |  |  |  |  |  |  |
| Tree pollen, total (grain/m^3^) | 0.0 | 0.0 | 0.0 | 2.2 | 3.6 | 4.4 | 8.6 |
| Oak tree pollen (grain/m^3^) | 0.0 | 0.0 | 0.0 | 0.0 | 0.0 | 0.0 | 0.0 |
| Grass pollen (grain/m^3^) | 0.0 | 1.4 | 2.2 | 4.3 | 6.6 | 9.6 | 19.9 |
| Ragweed pollen | 0.0 | 0.0 | 3.5 | 15.7 | 34.5 | 46.5 | 76.2 |
| Total weed pollen (grain/m^3^) | 4.4 | 10.8 | 17.5 | 33.5 | 59.2 | 77.0 | 113.6 |
| Birch pollen (grain/m^3^) | 0.0 | 0.0 | 0.0 | 0.0 | 0.0 | 0.0 | 0.8 |
| Molds (spore/m^3^) | 2184.7 | 3305.1 | 3959.3 | 4764.5 | 5813.4 | 6344.3 | 8341.5 |
|  |  |  |  |  |  |  |  |
| Rhinovirus | 58.0 | 80.0 | 88.0 | 151.0 | 174.0 | 193.0 | 256.0 |
| Respiratory syncytial virus | 2.0 | 3.0 | 5.0 | 7.0 | 8.0 | 15.0 | 19.0 |
| Influenza-A | 0.0 | 0.0 | 0.0 | 0.0 | 1.0 | 1.0 | 1.0 |
| Influenza-B | 0.0 | 0.0 | 0.0 | 1.0 | 1.0 | 1.0 | 3.0 |


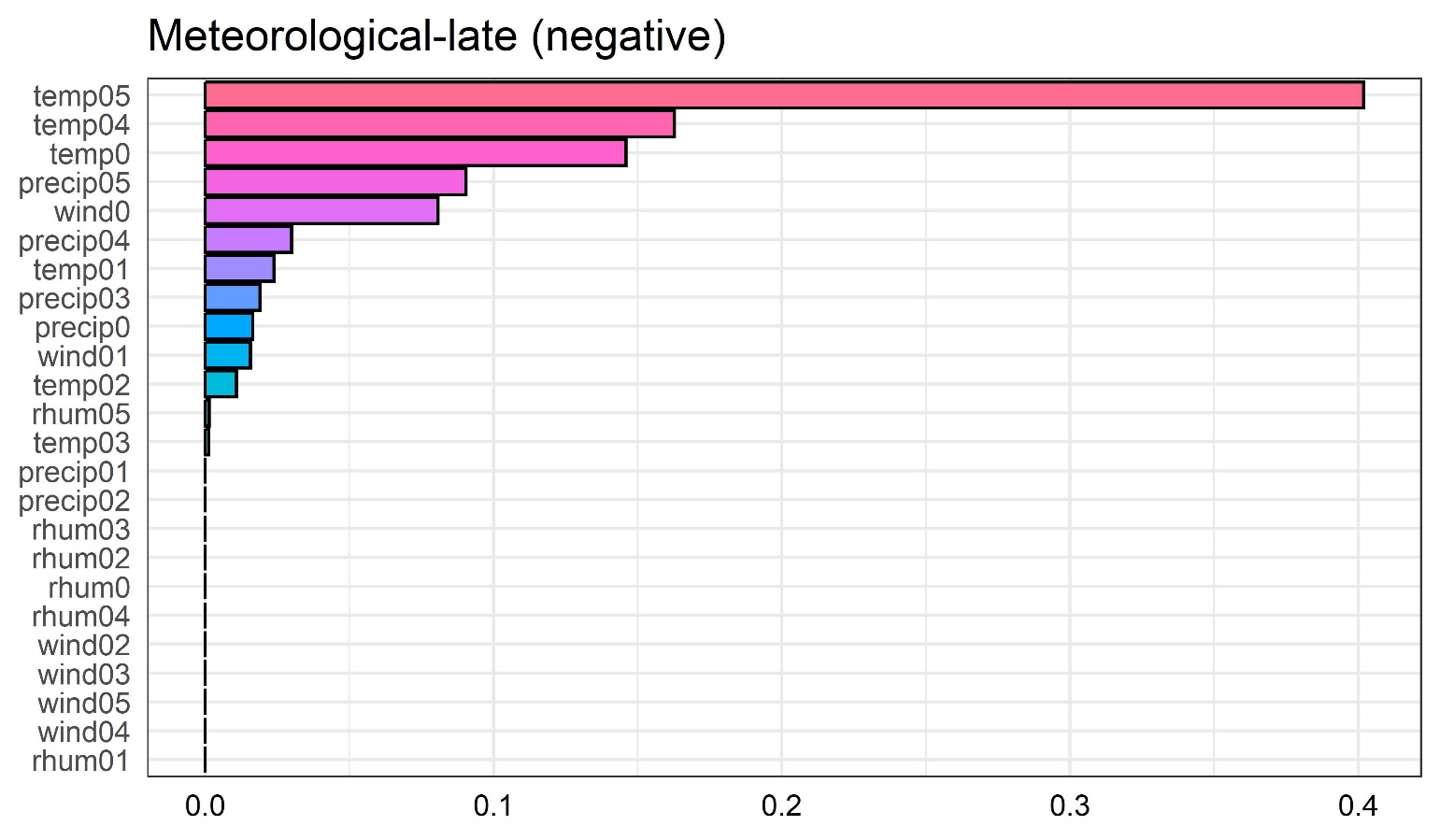


**Supplementary Fig.2.** **Relative importance of predictors within the *late pollen season*, as indicated by weights estimated from the generalized quantile weighted sum (gQWS) model, among predictor variables of meteorological factors (overall negative relationship with daily childhood asthma exacerbation counts).**


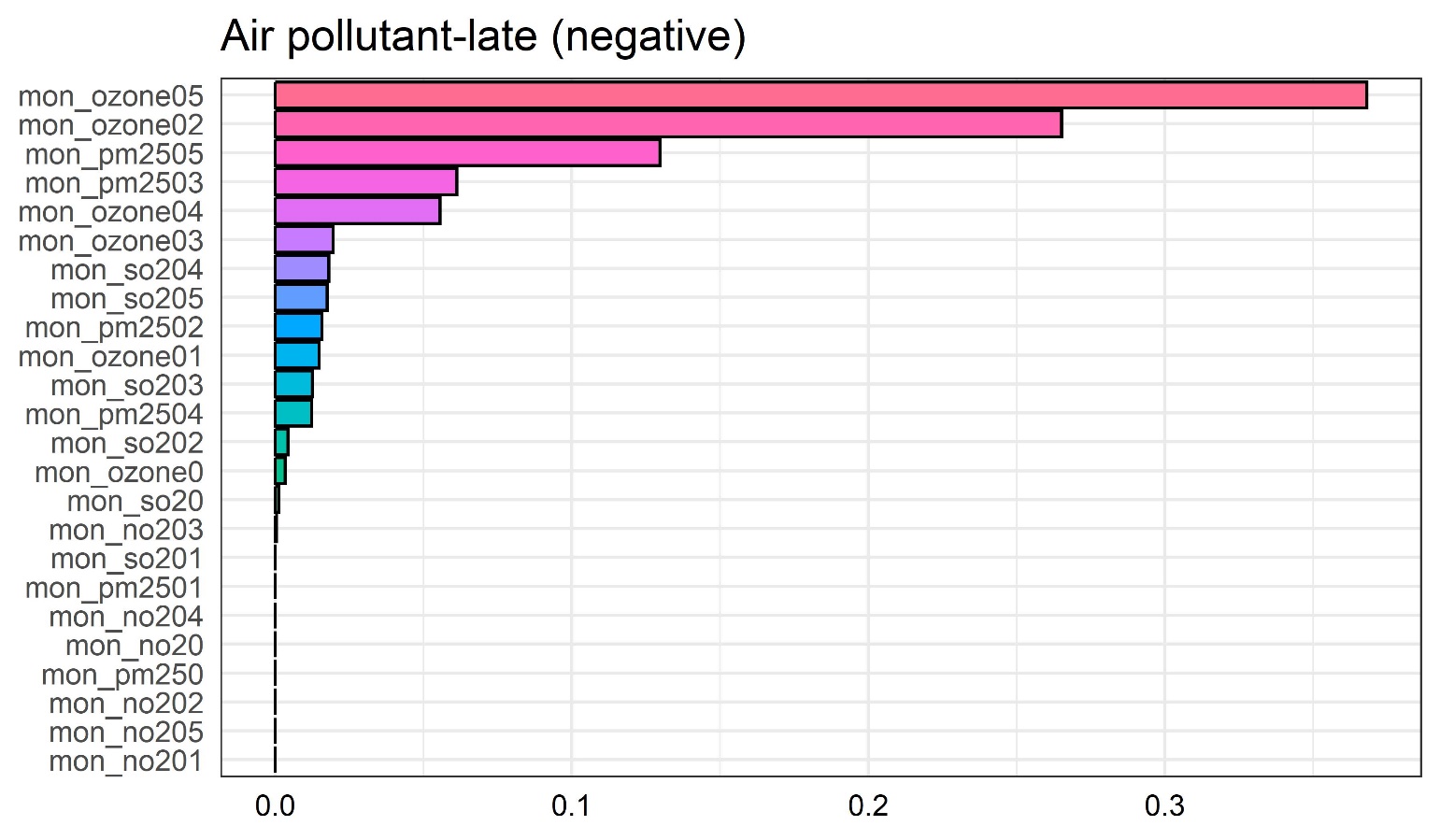


Supplementary Fig.3. Relative importance of predictors within the late pollen season, as indicated by weights estimated from the generalized quantile weighted sum (gQWS) model, among predictor variables of air pollutant factors (overall negative relationship with daily childhood asthma exacerbation counts).

Supplementary Table 4. Selected predictors (meteorological factors, air pollutants, aeroallergens, detected respiratory virus infections), determined based on 80 percent of cumulative weight from the gWQS regression, within the early pollen season (March 18 to June 30) from each study year (2011 to 2016).

| Predictor type | Predictor (lag) | lag0 | lag01 | lag02 | lag03 | lag04 | lag05 |
| --- | --- | --- | --- | --- | --- | --- | --- |
| Meteorological | Temperature |  |  |  |  | X(-) | X(-) |
|  | Relative humidity |  |  |  |  |  | X(-) |
|  | Wind |  |  |  |  | X(-) |  |
|  | Precipitation |  |  |  |  |  | X(-) |
|  |  |  |  |  |  |  |  |
| Air pollutants | PM_2.5_ |  |  |  | X(-) | X(-) | X(-) |
|  | O_3_ | X(-) | X(-) |  |  |  | X(-) |
|  | NO_2_ | X(+) |  |  |  |  | X(+) |
|  | SO_2_ |  | X(+) |  |  |  | X(+) |
|  |  |  |  |  |  |  |  |
| Aeroallergens | Tree pollen, total | X(+) |  |  | X(+) | X(+) |  |
|  | Oak pollen |  |  |  |  |  |  |
|  | Birch pollen | X(+) |  | X(+) |  |  |  |
|  | Grass pollen |  |  |  |  |  |  |
|  | Weed pollen, total |  |  |  |  |  |  |
|  | Ragweed pollen | X(-) | X(-) | X(-) | X(-) | X(-) | X(-) |
|  | Molds | X(-) | X(-) | X(-) |  |  | X(-) |
|  |  |  |  |  |  |  |  |
| Respiratory virus | Corona | X(+) |  |  |  |  |  |
|  | rhino |  |  |  |  |  |  |
|  | para1 | X(+) |  |  |  |  |  |
|  | para2 | X(+) |  |  |  |  |  |
|  | para3 | X(+) |  |  |  |  |  |
|  | Adeno |  |  |  |  |  |  |
|  | RSV |  |  |  |  |  |  |
|  | IFV-A | X(+) |  |  |  |  |  |
|  | IFV-B | X(+) |  |  |  |  |  |
|  | hMPV |  |  |  |  |  |  |

X: selected predictor; XX: forced-in selected predictor.

Within parentheses following each selected predictor, '+'/'-' indicates that the predictor was within a 'mixture' with overall positive/negative relationship to childhood asthma exacerbation.

^a^Meteorological, air pollutant, and aeroallergen predictors were all daily-level variables (with daily-level cumulative lags).

^a^Only same-month respiratory virus predictors were considered.

**Supplementary Methods Text**

*ARIMA model – parameterization*

We determined the parameters for ARIMA models, to capture the long-term trend and seasonal component, and therefore other (viral, environmental) predictors could explain the rest of the variations. Each ARIMA model (p,d,q)_s_ incorporates three parameters – with ‘p’ and ‘q’ describing the auto-regressive (AR) and moving-average components (MA) (i.e., the dependency between daily numbers of exacerbation cases); and the ‘d’ describing the level of differencing – a process transforming a non-stationary time series into a stationary one, in satisfaction of the stationary assumption for ARIMA models (i.e., the ‘d’ parameter captures the long-term trend of the time series, describing the numbers of childhood asthma exacerbation case occurrences each day during the study period). An additional term, ‘S’, denoted seasonality (227-days of the study period within each year [115 early-season days/112 late-season days].

*Generalized quantile weighted sum regression (gQWS) models for predictor selection*

To determine lagged predicators to be entered into a multivariable ARIMA model, we used generalized quantile weighted sum regression (gQWS). gWQS is a generalized linear model framework, in which a specific health outcome of interest is regressed upon a set of predictors (e.g., environmental exposures) to determine their relative importance (i.e., weights) {Carrico, 2015 #22}. Specifically, the framework allows the calculation of a weighted quantile sum index (WQS), upon which a regression coefficient is fit to maximize the likelihood of the model. Within the QWS term, the weight for the quantile sum of each predictor is averaged across all estimates from a series of bootstrapping (sub)samples and is constrained from 0 to 1 (with a cumulative sum of 1 of all predictors). We determined to use gQWS for predictor selection, as it has been suggested that gWQS is likely to out-perform individual regression and regularization methods (e.g., lasso, elastic nets), especially when the correlations between the predictors are relatively high (e.g., between different pollutants, different lagged exposure for a specific predictor). In fact, simulation studies have shown that traditional regularization methods were more likely to identify certain groups of correlated risk factors to be of high importance, upon shrinking the coefficients towards certain values {Carrico, 2015 #22}. Besides, unlike principal component analysis (PCA), the application of gQWS allows the relationship between asthma exacerbation and different risk factors to be taken into consideration, when selecting the predictors. With gQWS, a recent epidemiologic study examined the overall effects of air pollutant mixtures (i.e., air pollutants, aeroallergens) and pediatric asthma exacerbations, as well as the relative importance of each pollutant in the mixture {Puvvula, 2022 #31}. More specifically, we applied gQWS to determine a primary set (subset) of predictors was for each exposure group (respiratory viral infection, meteorological, air pollutants, aeroallergens), within the early- and late-aeroallergen season, respectively. To carry out this approach, the outcome of daily asthma exacerbation counts (assumed to follow a quasi-Poisson distribution) was regressed on each of the four different types of environmental and viral predictors (i.e., meteorological factors, air pollutants, aeroallergens, and respiratory viruses) as specified above in the **respiratory viral infection data** and **environmental data** sections, with each predictor spanning cumulative exposure latencies from lag0 to lag05. Each predictor was entered at a quartile scale (rather than linear) to allow a more flexible relationship with the asthma exacerbation outcome. And we entered viral infection and aeroallergen variables into the model first, due to the small number of days when both aeroallergen and respiratory viral infection levels were either high or low. We constructed a separate gQWS model, for each type of predictor, respectively – where all predictors belonging to that a certain type of environmental (air pollutant, aeroallergens, meteorological factors)/viral predictor set were considered as an integrated group of exposure. Due to the constraint of weight for each predictor from 0 to 1, the overall association between all the predictors in the gQWS model and childhood asthma exacerbation had to be assumed as positively and/or negatively associated with the outcome. Therefore, we attempted to fit two gQWS models for predictor group assuming either positive/negative associations, during the early- and late-season, respectively. In each fitted gQWS model that was able to run, a weight (as a percentage) was generated to indicate the relative importance of each predictor within that certain group of predictors, with cumulative weights of all the predictors in that model summed up to 100 percent. The relative importance of each predictor variable within that certain type of predictor was made comparable to all the others within each predictor group, with a higher weight indicating a higher relative importance. With the relative importance, we were able to determine a subset of predictors from the model for each predictor type – by sorting the weights of all predictors in descending order, retaining only a subset of predictors with 80 percent of cumulative weights – an arbitrary percentage cutoff determined *a priori*. If any gQWS model constructed for a certain group of predictors were able to run under both the positive and negative assumptions, then we ran both models in identification of the primary predictor set for that specific predictor type. Of note, total tree pollen levels were determined a priori as an important predictor, due to its substantial contribution to aeroallergen levels in the ambient environment, and multiple studies indicating increased exacerbation risk from tree pollen exposure {De Roos, 2020 #19}{Dales, 2008 #23}.

We attempted to fit gQWS models for each predictor group under both the assumptions of overall positive and negative associations between predictors and the outcome. Yet, gQWS models were able to be fit for some of the predictor groups with the assumption of both overall positive and negative associations for each in their relationship with childhood asthma exacerbation, whereas for other predictor groups gQWS models were only able to be fit under the assumption of either positive or negative overall relationships, but not both. Of note, to ensure model convergence, aeroallergen predictors were estimated under the assumption of an overall positive relationship with asthma exacerbation as an entire predictor group in the gQWS regression.
